# Sperm physiology varies according to ultradian and infradian rhythm**s**

**DOI:** 10.1038/s41598-019-42430-4

**Published:** 2019-04-12

**Authors:** Ayelén Moreno-Irusta, Jackelyn M. Kembro, Esteban M. Domínguez, Arturo Matamoros-Volante, Maria N. Gallea, Rosa Molina, Hector A. Guidobaldi, Claudia L. Treviño, Maria J. Figueras, Ana Babini, Nelso A. Paina, Carlos A. N. Mercado, Laura C. Giojalas

**Affiliations:** 1 Universidad Nacional de Córdoba (UNC), Facultad de Ciencias Exactas, Físicas y Naturales, Centro de Biología Celular y Molecular, Córdoba, Argentina; 2 Instituto de Investigaciones Biológicas y Tecnológicas, UNC, CONICET, FCEFyN, Córdoba, Argentina; 3 Departamento de Genética del Desarrollo y Fisiología Molecular, Instituto de Biotecnología, Universidad Nacional Autónoma de México, Cuernavaca, Mexico; 4 Laboratorio de Andrología y Reproducción (LAR), Córdoba, Argentina; 5 Universidad Nacional de Córdoba, Facultad de Ciencias Médicas, Instituto Universitario de Medicina Reproductiva (IUMER), Córdoba, Argentina; 6 Universidad Nacional de Córdoba, Hospital Universitario de Maternidad Nacional, Córdoba, Argentina

## Abstract

The spermatozoon must be physiologically prepared to fertilize the egg, process called capacitation. Human sperm samples are heterogeneous in their ability to capacitate themselves, which leads to variability between samples from the same or different donors, and even along the seasons. Here we studied sperm variation in the capacitation state according to the ability of capacitated spermatozoa to acrosome react upon stimulation (% ARi) and to be recruited by chemotaxis (% Chex). Both indirect indicators of sperm capacitation increased along the incubation time with fluctuations. Those capacitated sperm recruited by chemotaxis showed an ultradian rhythm with a cycle every 2 h, which might be influenced by unknown intrinsic sperm factors. Two infradian rhythms of 12 months for the % ARi and of 6 months for % Chex were observed, which are associated with the joint action of temperature and photoperiod. Thus, to avoid false negative results, human sperm samples are recommended to be incubated for a long period (e.g. 18 h) preferably in spring time. This innovative point of view would lead to better comprehend human reproductive biology and to think experimental designs in the light of sperm cyclicity or to improve sperm aptitude for clinical purposes.

## Introduction

The spermatozoon must be in such a physiological state to guarantee not only fertilization but also early embryo development^1^. This physiological preparation is acquired after a period of incubation under adequate physical and chemical conditions. This process is generally known as sperm capacitation^2,3^, comprising a series of biochemical and biophysical changes occurring at the sperm plasma membrane and also inside the cell. For instance, during capacitation, changes have been reported such as an increase in intracellular pH^4,5^, calcium^6^, reactive oxygen species (ROS)^7^, cAMP and protein tyrosine phosphorylation^8^. Once the spermatozoon is capacitated, it can undergo several sperm processes that help successful fertilization, such as the induced acrosome reaction^9^ and the orientation of its movement by following the concentration gradient of an attractant molecule, known as chemotaxis^10,11.^

The timing and incubation conditions for sperm capacitation vary according to the species, but also between laboratories. In the case of human spermatozoa, even though most researchers include the essential components to support capacitation in the culture medium (calcium, bicarbonate and albumin)^12^, the timing differs greatly between labs, from two hours to overnight incubation. There is also great variation between semen samples from the same and different donors^13^. Human sperm variations may be attributed, among other factors, to a seasonal change in climate, especially in regions with marked seasonality, but such reports are controversial. While some groups reported variation in spermogram parameters during the year^14–18^ others did not observe any seasonal trends^19–21^. These differences could be due to sample size and method of analysis. Moreover, it is worth noting that most of the semen parameters analyzed (i.e. sperm concentration, motility and morphology) are still controversial as predictors of male fertility. Parameters more representative of the global physiological state would be preferable, for example sperm capacitation^22,23^. To verify whether sperm capacitation varies with some kind of periodicity, e.g., less than 24 h (ultradian rhythm) or higher than 24 hours (infradian rhythm, which includes periods of one year, i.e. a circannual rhythm), robust mathematical approaches should be applied as those used to determine cyclicity in epidemiological events^24^.

Since our laboratory is in the city of Córdoba (Argentina), which is immersed in a humid subtropical climate with marked seasonality, we investigated whether sperm capacitation varies according to an ultradian and/or infradian rhythm, with the aim of explaining sperm physiology variations.

## Results

### Sperm physiology ultradian variation

We first evaluated the temporal dynamics of the capacitation state in the same sperm sample, every hour, during 24 h incubation, by means of the % AR_i_^12^ and % Chex spermatozoa^25^.

**Figure 1:**
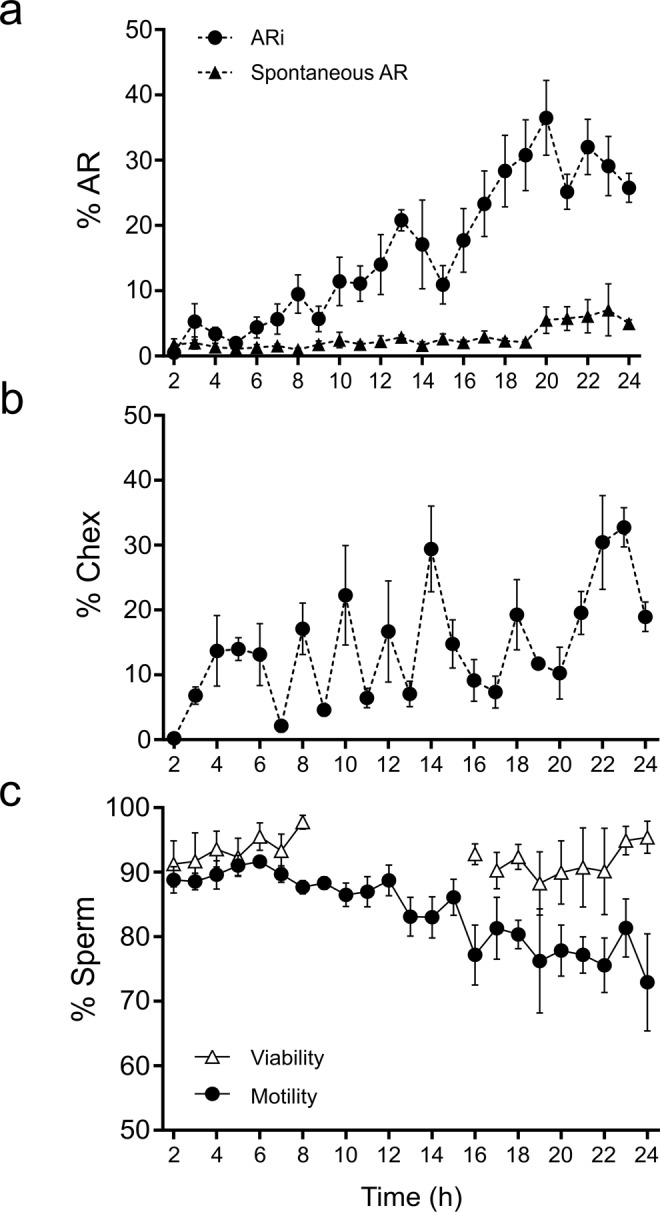
Sperm physiology fluctuation along incubation time. Percentage of AR_i_ spermatozoa (circles) and the percentage of spontaneous AR spermatozoa (triangles) during 24-hours incubation period under capacitating conditions (**a**). Percentage of Chex during 24-hours incubation period under capacitating conditions (**b**). Percentage of motile (black circles) and live (white triangles) spermatozoa, during 24 hours incubation under capacitating conditions (**c**). ARi, induced acrosome reaction; Chex, capacitated spermatozoa recruited by chemotaxis. Data are expressed as the mean ± SEM, of 8 independent samples.

**Figure 2:**
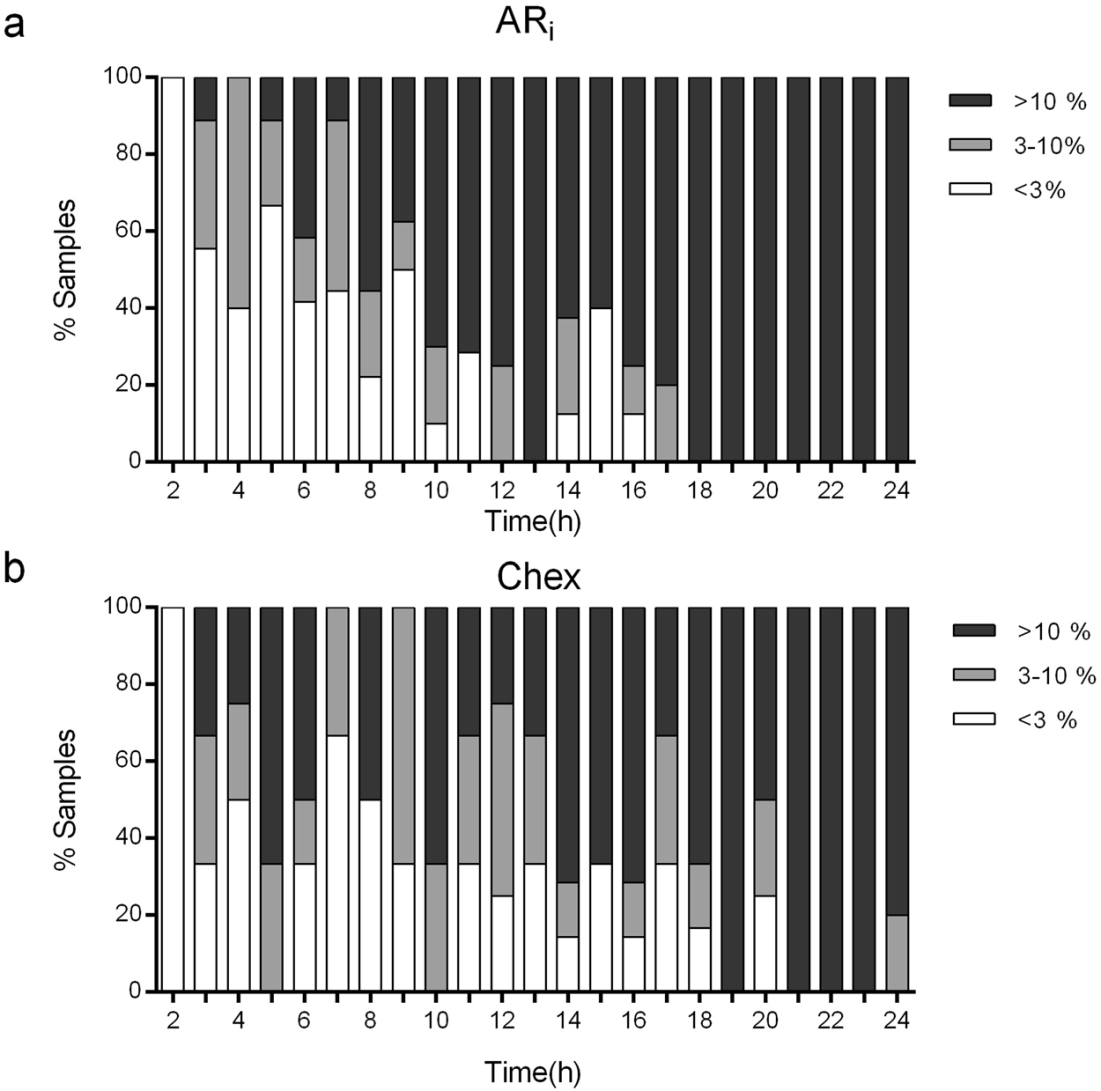
Distribution of sperm samples according to the physiological state. Percentage of samples showing a defined range of % AR_i_ (**a**) or % Chex (**b**), determined during incubation. The corresponding ranges of each parameter are shown in the upper right corner of each Figure. AR_i_, induced acrosome reaction; Chex, capacitated spermatozoa recruited by chemotaxis. Data include measurements of 9 independent experiments performed with ejaculates from different donors.

The mean % AR_i_ (Fig. 1a) and % Chex spermatozoa (Fig. 1b) gradually increased with fluctuations as a function of incubation time. Interestingly, the percentage of sperm samples showing a defined range of capacitation (either AR_i_ or Chex) also varied during incubation. For both parameters, 100% of the semen samples showed a value lower than 3% after 2 h incubation, in contrast to the 70–100% of the samples that achieved a value greater than 10% after 18 h incubation (Fig. 2).

**Figure 3:**
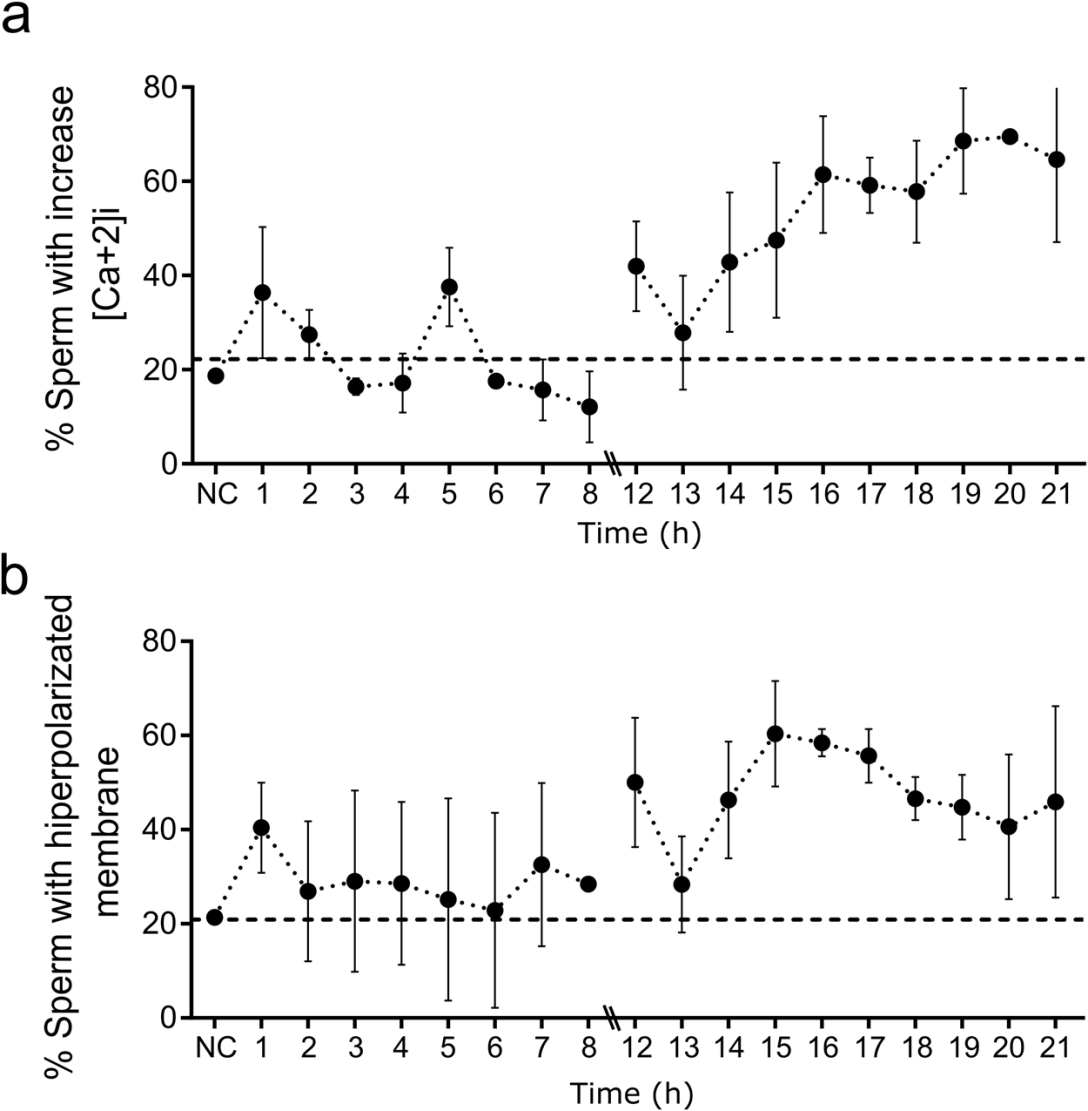
Sperm membrane potential and intracellular Ca^+2^ along incubation time. Percentage of spermatozoa showing an increase in: the intracellular calcium (**a**) and the hyperpolarized membrane (**b**). For the analysis, the percentage of spermatozoa exceeding the third quartile of fluorescence intensity was taken; the dotted line shows the percentage of spermatozoa that exceeds the third quartile in non-capacitated samples (NC). Data are expressed as the mean ± SEM of 8 independent experiments performed with ejaculates from different donors.

In addition, the intracellular calcium and the hyperpolarization of the sperm membrane (parameters associated with sperm capacitation) apparently increased during incubation compared with non-capacitated spermatozoa (Fig. 3). Moreover, the increase in sperm capacitation over time was verified by protein tyrosine phosphorylation (Supplementary Fig. S1). As expected, the spontaneous AR (Fig. 1a), and sperm motility and viability (Fig. 1c) remained quite stable throughout incubation.

**Figure 4:**
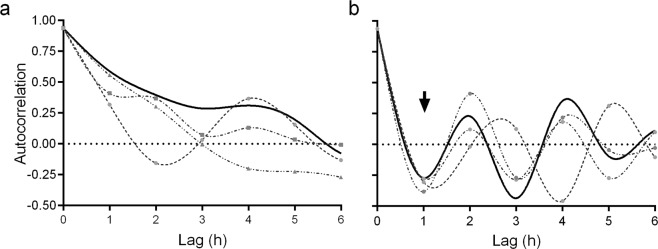
Verification of cyclicity in sperm physiology along incubation time. Autocorrelation analysis of time series of three representative samples (gray lines) and the average of all the samples (N = 7; black line) from data represented in Fig. 1, determined as: % AR_i_, (**a**) and % Chex (**b**) during 24-hours incubation period. Black arrow points to a negative autocorrelation value (average curve) that is followed by a positive one, indicating that overall at 1-hour intervals higher values are followed by lower values, while the lower are followed by larger values, indicating the existence of periodicity. ARi, induced acrosome reaction; Chex, capacitated spermatozoa recruited by chemotaxis.

The occurrence of peaks and valleys in the % AR_i_ and % Chex spermatozoa was also observed in individual samples (Supplementary Fig. S2). Therefore, the fluctuations observed may be due to a random phenomenon or to sperm capacitation periodicity (i.e., peaks recurring at a certain time lag). To elucidate the nature of the variation, we studied the presence of ultradian rhythms (less than 24 h) by autocorrelation analysis of time series. The % AR_i_ spermatozoa showed an exponential decay in the autocorrelation function for lags up to 4 h (Fig. 4a), indicating that variations in AR_i_ may represent a stochastic process with short-range correlations. In contrast, the mean % Chex spermatozoa showed a cyclic fluctuation with peaks and valleys, with a period of approximately 2 h (Fig. 4b), as shown by the periodic behavior observed in the autocorrelation function, with negative values followed by positive values at an interval of 2 h (Fig. 4b). This cyclic behavior was also seen in individual sperm samples (Fig. 4b; Supplementary Fig. S2). Our results indicate that both parameters increase with time, and that only the % Chex spermatozoa fluctuate with an ultradian rhythm with a 2-hour cycle.

### Sperm physiology infradian variation

**Figure 5:**
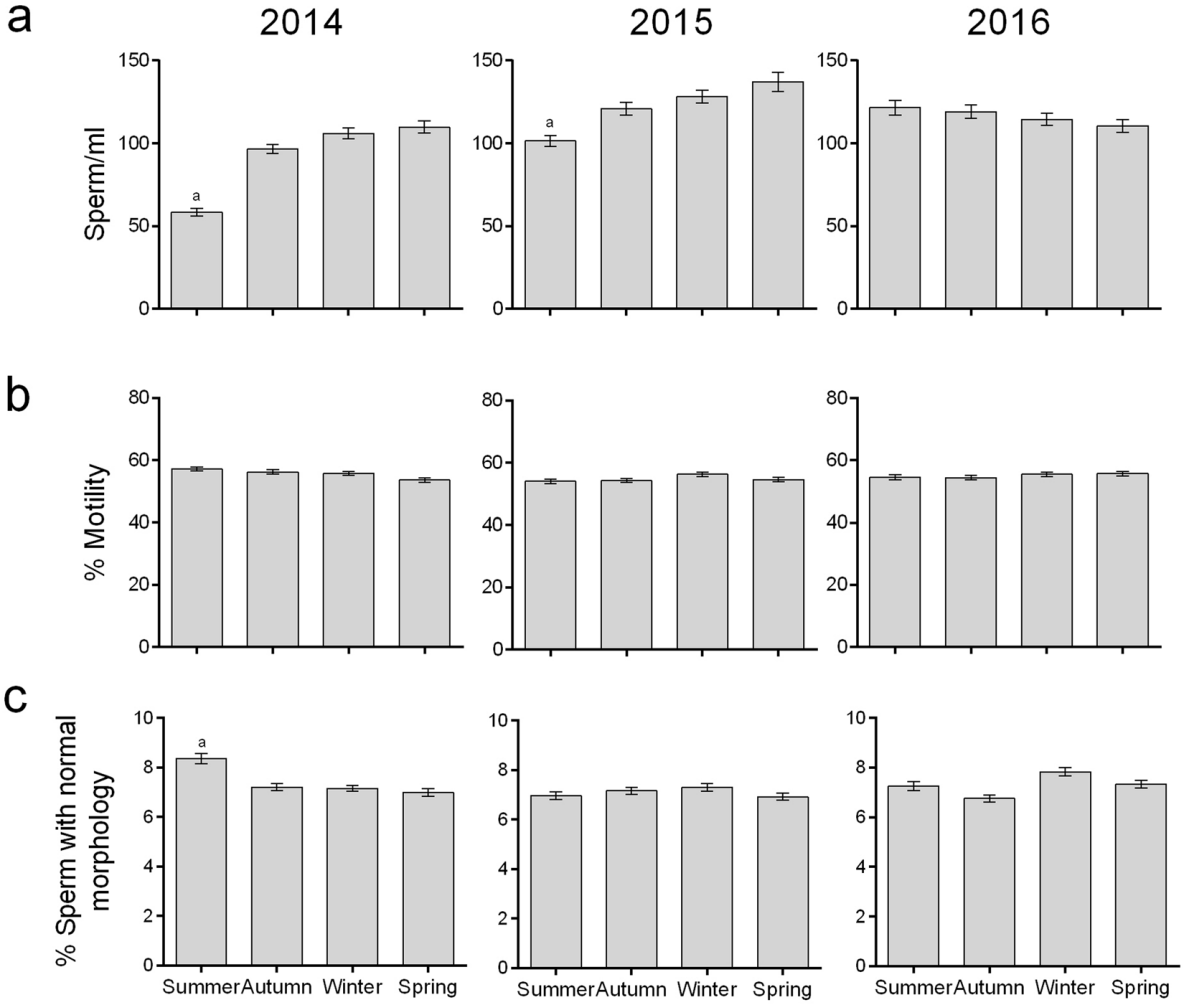
Sperm concentration, motility and morphology values along a three-year period (2014–2016). Sperm concentration (**a**). Percentage of motile sperm (**b**). Percentage of spermatozoa with normal morphology (**c**). Data are expressed as the mean ± SEM of N ∼ 1400 samples per year. ^a^Statistically significant differences vs all the other categories.

**Figure 6:**
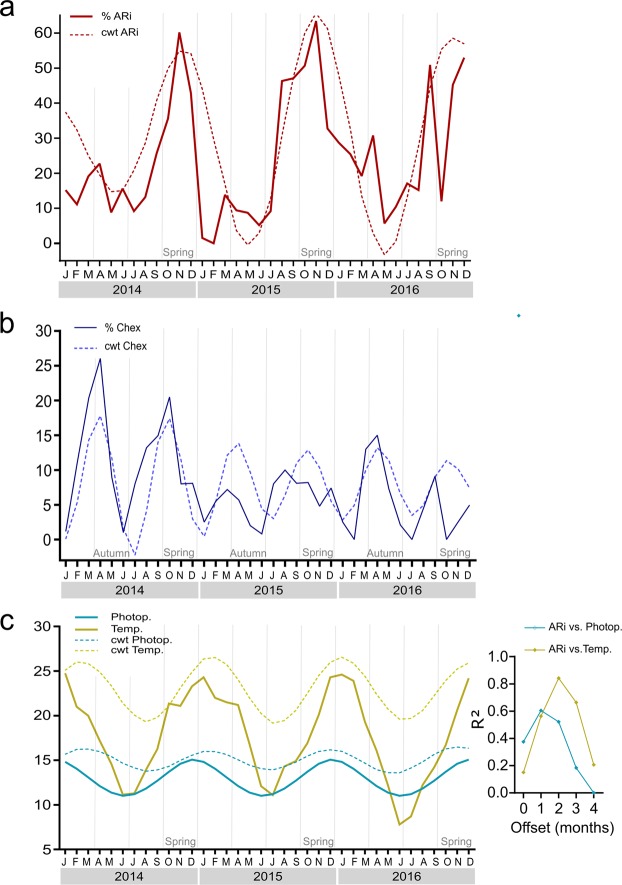
Infradian rhythms observed in sperm physiology after 18 h incubation and its relation to environmental factors. The time series of the % AR_i_ sperm and the % Chex sperm are shown in panel (a,b), respectively, while photoperiod (light blue) and mean temperature (yellow) time series (continuous lines) are shown in panel (c). The corresponding Morlet Wavelet coefficients (cwt) at different monthly scale (dotted lines) are shown in each panel. Vertical gray lines indicate the change in season. Inset corresponds to the correlation coefficient (R^2^) obtained between Morlet Wavelet coefficient at a 12-month scale of % AR_i_ and photoperiod (blue) or temperature (yellow), at different offsets. To improve visualization, Wavelet coefficients were offset by values of 30, 18, 15 and 10 for % ARi, temperature, photoperiod, and %Chex, respectively. Data are expressed as the mean of 3–4 independent experiments, for each month, performed with ejaculates from different donors. Temperature and photoperiod are shown as the mean of data from 36 consecutive months. ARi, induced acrosome reaction; Chex, capacitated spermatozoa recruited by chemotaxis; cwt, wavelet coefficents; Photop., photoperiod; Temp., temperature.

**Figure 7:**
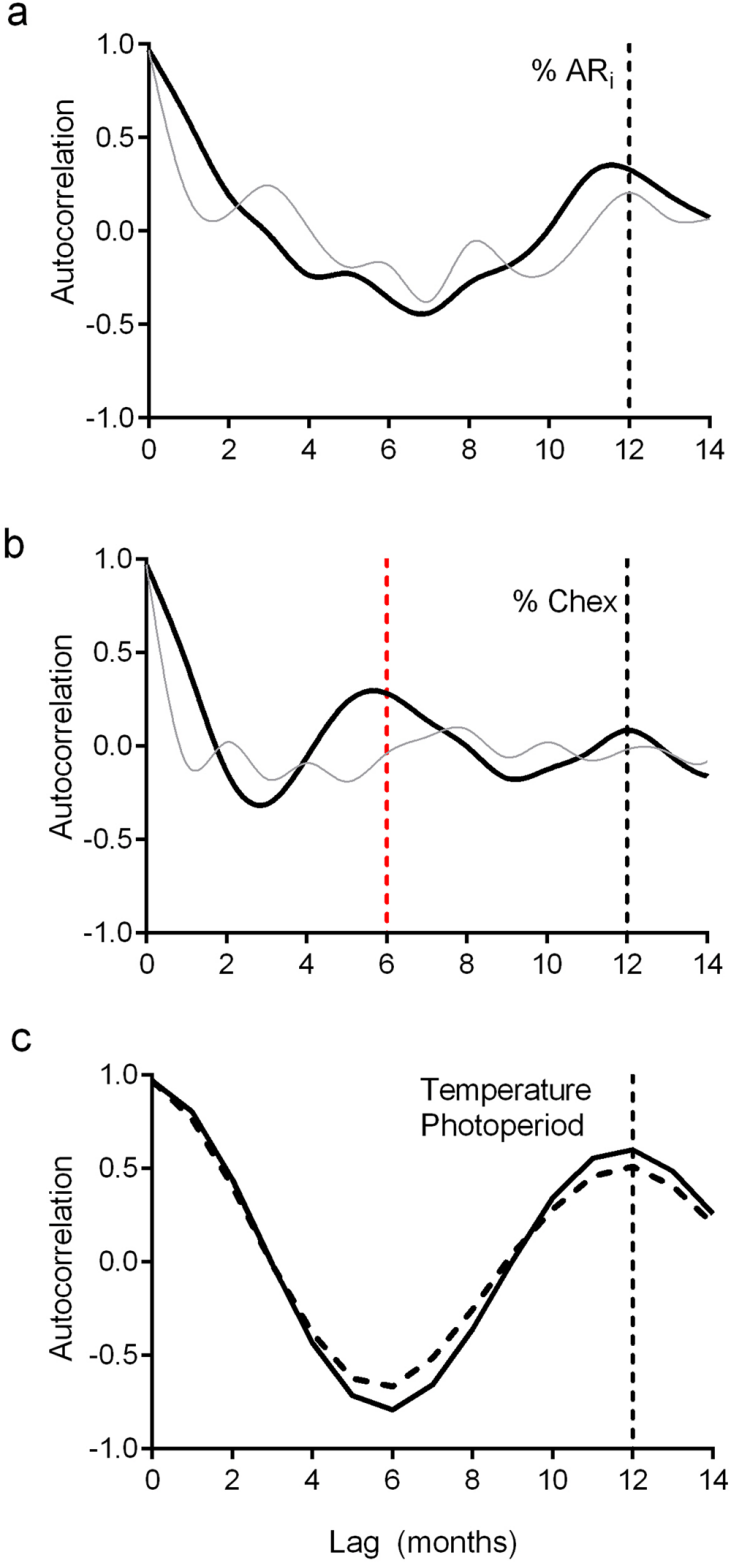
Autocorrelation analysis for sperm capacitation over a 3-year period. Autocorrelation analysis of the % AR_i_ (**a**) or % Chex (**b**) spermatozoa incubated under capacitating conditions for 4 h (gray line) and 18 h (black line), and the average temperature (gray solid line) and photoperiod (dotted line) values (**c**). Time series used in this analysis are based on data from Fig. 6. Red and black dotted lines highlight the 6 and 12month cycle, respectively. ARi, induced acrosome reaction; Chex, capacitated spermatozoa recruited by chemotaxis.

We next studied whether conventional semen parameters (concentration, morphology and motility) vary or not over the seasons. The three parameters, provided by a local andrology lab, seem to behave quite stable throughout the year, except for a decrease in sperm count in summer time in two of the three years (Fig. 5). However, since the autocorrelation coefficient approached zero, seasonal periodicity is not evident, (Supplementary Fig. S3); hence, we next looked for seasonality in sperm capacitation. The percentages of AR_i_ and Chex spermatozoa were determined in the middle of each month, and the cells incubated under capacitating conditions for 4 h (incubation time chosen by many scientists) and 18 h (time at which most of the samples show a capacitation level higher than 10% in this study), during three consecutive years (from 2014 to 2016). From visual observation of the time series of % AR_i_ and % Chex after 18 h of incubation, an apparent infradian variation over the years is observed (solid line in Fig. 6a,b). Moreover, for both sperm parameters, the three year mean value for peaks was significantly higher than that observed in valleys (% AR_i_: 46 ± 6% vs 12 ± 3%; p < 0,001; % Chex: 12 ± 2% vs 5 ± 1%; p < 0.0002). A similar behavior was observed after 4 h of incubation (solid line in Supplementary Fig. S4). To verify the existence of a circannual variation, an in-depth mathematical analysis was performed. Thus, the autocorrelation analysis for the % AR_i_ showed negative values at 6 months followed by positive values at 12 months, reflecting variations for periods of one year (Fig. 7a). A similar result was obtained with the Wavelet analysis run at different time scales. We found that the scale of 12 months shows periodicity according to the variations observed in the alternation of red (high cwt) and blue (low cwt) patterns (Supplementary Fig. S5) where the corresponding wavelet coefficients are shown as peaks and valleys in Fig. 6 (dotted lines), hence, % AR_i_ varies with a circannual rhythm with a peak in spring time. These results were further verified by the complex Gaussian wavelet (Supplementary Fig. S5). Even though the level of Chex seems also to vary along the year, its periodicity is apparently different from that shown by the AR_i_ (Fig. 7b). Thus, the three periodicity tests showed an infradian cycle that is repeated every 6 months, with peaks in autumn and spring (Figs 6b and 7b, Supplementary Fig. S5).

In addition, we mathematically verified the yearly variations of temperature and photoperiod, parameters that govern seasonality in our region. As expected, the autocorrelation and Wavelet analysis show a circannual periodicity in these parameters (Figs 6c and 7c, Supplementary Fig. S5). We next investigated whether the periodicity of these meteorological parameters was correlated with those linked to sperm capacitation. High levels of correlations are observed between the Wavelet coefficients of the AR_i_ and meteorological parameters. Thus, the peak of % ARi precedes in 1 or 2 month those of photoperiod and temperature, respectively (inset Fig. 6c). It is worth to note that a similar correlation analysis cannot be performed between % Chex and meteorological parameters since they do not share the same periodicity. These results show the existence of seasonal variation in the sperm capacitation state, at least as indirectly evaluated as the percentages of AR_i_ and Chex spermatozoa.

## Discussion

Here we show that sperm physiology varies with different rhythms observed at different time scales: ultradian (a 2 h cycle for % Chex sperm) and infradian (a 6-months cycle for % Chex sperm and a 12-month cycle for % AR_i_ sperm).

Both indicators of sperm capacitation increase along time with a fluctuating pattern of peaks and valleys. The % AR_i_ showed no periodicity, while the % Chex expressed a well-defined cycle of approximately 2 hours, consistent with the notion that the capacitation state is transient and lasts for 2 hours^26,27^. The different behavior of these two parameters may reflect technical influence, since it is probable that post-capacitated spermatozoa (those that lost the capacitation state) also acrosome react upon pharmacological induction^28,29^, while only capacitated acrosome-intact spermatozoa are able to respond to a chemoattractant stimulus^11,30,31^. It is worth noting that high levels of motility and viability are observed throughout the 24-hour incubation period, suggesting that the observed variations are preferably associated to changes in the capacitation state. In addition, other parameters associated with sperm capacitation, such as the intracellular level of calcium and the hyperpolarization of the sperm membrane^13^ increased along incubation but with apparent mild fluctuations.

Interestingly, all the sperm samples incubated for only 2 hours showed negligible capacitation (with % AR_i_ and % Chex values less than 3%), in contrast to most of the sperm samples that obtained a ≥10% level after 18-hour incubation period. Thus, a long incubation ensures that most of the samples acquire the capacitation state, at least in terms of induced acrosome reaction and sperm recruitment by chemotaxis.

Sperm physiology variation may also be associated with an infradian rhythm. Several laboratories investigated seasonality in seminal parameters, since they are a direct consequence of spermatogenesis and epididymis maturation. However, seasonality in sperm concentration, morphology and motility parameters is controversial^14,18,32^. Here we show that these sperm parameters do not behave with an infradian rhythm.

After evaluating sperm capacitation, every month for three years, we concluded that the sperm samples show a different periodicity according to the parameter evaluated. Thus, the % AR_i_ has a 12-month rhythm with a peak in spring (October-November), meanwhile the % Chex shows a 6-month rhythm, in which, in the first semester of the year, the peak occurs in autumn (March-April), and in the second semester the peak is observed in spring (September-October). The latter matches the peak of the % AR_i_ and that of meteorological parameters. In other words, the time of the year where the level of capacitation is best is in spring.

Which factors may influence the seasonality observed in sperm physiology? Given that temperature and photoperiod are highly correlated, it is difficult to dissociate their influences on sperm physiology. Moreover, both are quite well correlated to the % AR_i_ during the year, while the second peak of the year of the % Chex also matches the increase in meteorological parameters. However, the Chex peak in the first semester of the year seems to be modulated by other unknown factors. The present work shows that the capacitation level is very low in summer and winter; but this may be due to a direct effect of environmental factors on the physiology of spermatozoa or to an indirect effect on spermatogenesis. For instance, the biological influence of photoperiod may reside in that it modulates the seasonal changes in night length, which is negatively correlated with the melatonin concentration in blood^33–36^. At the cellular level, the biological clock that regulates the cyclicity of somatic cell is governed by the expression of specific genes^37^. Since the spermatozoon is a transcriptionally inactive cell, another system may operate. For instance, the presence of melatonin in seminal plasma^38–40^ and the corresponding receptors on the sperm surface^36,39,41^ suggest that sperm physiology may be regulated by the melatonin circannual cycle. Indeed, exogenous melatonin administration enhances sperm function and ART results^42,43^.

Our results show that the circannual rhythm, and in part that of 6 months, may be modulated by the joint action of temperature and photoperiod, while the ultradian rhythm may be influenced by unknown intrinsic sperm factors. Thus, a long incubation (e.g. 18 h) for samples obtained preferably in spring time (in a seasonal climate) would be advisable to avoid false negative results. Here we present an innovative approach to better comprehend human reproductive biology to think experimental designs in the light of sperm cyclicity or to improve sperm aptitude for clinical purposes.

## Materials and Methods

### Ethics statement

The experiments were performed with human sperm samples provided by volunteer donors. The semen samples were treated in accordance with the Declaration of Helsinki. The study received the approval of the Ethics Committee of the National Clinical Hospital (National University of Cordoba, Argentina; # 061/10) and donors signed an informed consent document.

### Reagents and culture medium

All chemicals were purchased from Sigma-Aldrich (St. Lois, MO, USA) unless otherwise indicated. The non-capacitating medium used in this study was modified BWW, containing 120 mM NaCl, 4.8 mM KCl, 0.22 mM CaCl_2_, 1.16 mM MgSO_4_, 1.16 mM KH_2_PO_4_, 5 mM Glucose, 0.2 mM Na-Pyruvate, 11.7 mM Na-lactate, 45 mM Hepes, and 8% (w/v) Gentamicin. For capacitating conditions, the medium was supplemented with 25 mM NaHCO_3_, 1.68 mM CaCl_2_, and 3% (w/v) BSA, and osmolarity was adjusted by reducing the NaCl concentration to 88 mM. All media were adjusted to pH 7.4 and osmolarity was maintained around 290 mOs kg^−1^.

### Sperm preparation

Human sperm samples were collected by masturbation after 2–5 days of sexual abstinence. Only those samples exhibiting normal seminal parameters according to the WHO criteria^44^ were included in the study. Semen samples were liquefied at 37 °C under an atmosphere of 5% CO_2_ in air, for 30 min. Then, spermatozoa were separated from the seminal plasma by the migration-sedimentation technique^45^ or the swim-up procedure^46^, as specified in each set of experiments. In both cases, 1 ml of the semen sample was carefully placed beneath 1 ml of BWW capacitating or non-capacitating medium, and then the samples were incubated for 1 h at 37 °C under an atmosphere of 5% CO_2_ in air. At the end of incubation, the highly motile sperm population was recovered, and the sperm concentration was adjusted to 6 × 10^6^ sperm/ml in BWW medium, and further incubated with or without capacitation medium for different periods as specified in the Results section.

### Sperm capacitation evaluation

Since there is no available marker for sperm capacitation, we used several parameters that indirectly evaluate this physiological state.

#### Ability of capacitated spermatozoa to undergo the induced acrosome reaction

Briefly, the sperm sample was incubated with or without A23187 calcium ionophore (10 µM) for 30 min at 37 °C^12^. Then, the samples were fixed with 2% formaldehyde for 10 min at room temperature, centrifuged at 2000 g and washed twice with 100 mM ammonium acetate at pH 9.0. The pellet was resuspended in 0.2 ml of 100 mM ammonium acetate and 30 µl of the sperm suspension was smeared on a glass slide with the help of another glass slide. The sperm samples were let dry in air and then were incubated in freshly-made Coomassie Brilliant Blue stain (0.22% Coomassie G-250, 50% methanol, 10% glacial acetic acid, and 40% water), for 7 min at room temperature. Slides were thoroughly washed with distilled water to remove excess stain. After air drying, the status of the acrosome (intact or reacted) was evaluated in 200 cells, under bright field microscopy at 100× (*Olympus* BX 50, Center Valley, USA). The percentage of net induced acrosome-reacted spermatozoa (% AR_i_) was determined as the difference in the percentages of induced and spontaneous acrosome-reacted spermatozoa.

#### Recruitment of capacitated spermatozoa by chemotaxis

We applied the Sperm Selection Assay (SSA) device^25^, which consists of two wells, in which the sperm suspension is placed in one well (W1) and the attractant solution (or culture medium as negative control) in the other (W2). These two wells are connected by a tube, through which the attractant diffuses from W2 to W1, forming a concentration gradient which stimulates sperm chemotaxis. A 10 pM solution of progesterone was used as attractant^47^. The device was then incubated at 37 °C in an atmosphere containing 5% CO_2_ in air, for 20 min. At the end of the assay, the net percentage of sperm accumulation in W2 after the SSA (% Chex) was determined. This parameter was calculated as the difference in the percentages of spermatozoa recovered from W2 with or without progesterone.

#### Protein tyrosine phosphorylation pattern associated with the capacitation state

This parameter was determined with an imaging flow cytometer^48^. The samples were centrifuged at 2000 g for 3 min and the pellet was resuspended in 1 ml of PBS, adjusting the cell concentration to 10 × 10^6^ sperm/ml. Spermatozoa were fixed with 2% (v/v) paraformaldehyde in PBS for 20 min at room temperature, washed, and treated with 0.05% Triton X-100 in PBS for 15 min. Samples were then centrifuged at 4000 g in PBS for 5 min. The sperm pellet was resuspended in 1 ml of blocking solution (3% BSA in PBS) for 2 h at room temperature. The incubation with the first antibody (anti-phosphotyrosine monoclonal antibody clone 4G10, cat #05–321 from EMD Millipore, 1:500 dilution) was performed in 0.05% (v/v) Tween 20 in PBS (PBS-T), supplemented with 3% BSA, at 4 °C overnight. The sample was then centrifuged at 4000 g in PBS for 5 min and incubated with Alexa-488 conjugated anti-mouse IgG antibody (1:500) diluted in PBS-T, supplemented with 3% BSA, in the dark for 1 h at room temperature, performing the final wash with PBS. The control samples were processed as described above, but lacking either the specific or the second antibody, or both. The protein tyrosine phosphorylation labeling was determined in the sperm tail using an Image-based flow cytometer^48^. Briefly, the mask-based analysis allowed us to identify and quantify pY staining in the different sperm regions using an unbiased approach by quantifying pixel values. We applied the masks to all images and obtained fluorescence intensity values for the principal piece region of every cell (~2000 cells per experiment). Since fluorescence intensity varies among experiments, the comparison between treatments was performed by normalizing each group to the non-capacitating condition, performing the analysis with the percentage of spermatozoa exceeding the third quartile of fluorescence intensity.

### Sperm parameters associated with capacitation

Since a variation in the level of intracellular calcium and the membrane potential was observed during sperm capacitation^13^, these two parameters were also studied during incubation, and sperm viability was determined as an internal control.

#### Sperm intracellular calcium concentration, membrane potential and viability

These parameters were determined by flow cytometry, as previously described by Lopez-Gonzalez *et al*.^13^. Briefly, the sperm plasma membrane potential and [Ca^2+^] _i_ changes were monitored using the fluorescent indicators DiSC3(5) and Fluo3-AM, respectively. After incubation under either non-capacitating or capacitating conditions, samples were centrifuged at 750 g for 5 min. Spermatozoa were resuspended in BWW medium and the concentration was adjusted to 4 × 10^6^ sperm/ml. The sperm were loaded with 50 nM DiSC3(5) and 0.5 mM Fluo3-AM during 30 min at 37 °C under 5% CO_2_ in air, and after that the spermatozoa were washed by centrifugation. For each experimental condition, 500 µl of the cell suspension were placed in a cytometer tube, and 100 nM propidium iodide (PI) was used to determine sperm viability, added 1 min before collecting data.

Data were recorded as individual cellular events using a FACS Canto IITM cytometer (Becton Dickinson). Forward scatter (FSC) and side scatter (SSC) fluorescence data were collected from 20000 events per sample. Appropriate cytometer settings were selected for DiSC3(5), Fluo3-AM and PI. Threshold levels for FSC and SSC were set to exclude signals from cellular debris. DiSC3(5), Fluo3-AM and PI were excited using a 488-nm argon excitation laser. Nonviable cells became PI positive, and their red fluorescent signal detected as fluorescence of wavelength 670 nm. DiSBAC2(3)-positive cells were detected at 561–606 nm and Fluo3-AM-positive cells were detected at 515–545 nm. Unstained control samples were used to verify that threshold settings were appropriate and to create the corresponding gates needed to discriminate debris from cells. As a positive control, PI-stained dead sperm (sperm suspended in 0.1% Triton X-100 in BWW and incubated 10 min at room temperature) was run in parallel. Data were analyzed using FACS Diva and FlowJo software (Tree Star 9.3.3). The analysis was performed with the percentage of spermatozoa exceeding the third quartile of fluorescence intensity.

### Sperm motility

The percentage of motile spermatozoa was determined at the end of each incubation period by means of video microscopy and image analysis^49^, using a phase contrast microscope (Nikon Instruments Inc, NY, USA) and NIS elements software (4.30.01 DU1). The sperm movement from three different fields selected at random was digitally recorded at 30 Hz for 10 s under a 10x objective. Sperm tracks were analyzed by FIJI software using the motility tool plug-in, determining the percentage of motile cells based on motility of at least 200 spermatozoa.

### Sperm parameters database provided by a local andrology lab

Data from 4044 normospermic samples, corresponding to sperm concentration, motility and morphology, were determined by standard procedures according to WHO criteria^44^, and collected during three consecutive years (2014–2016) in a local Andrology lab, the standard quality of its procedures is certified by the Argentine Society for Reproductive Medicine.

### Meteorological data

The average temperature and the photoperiod were obtained from the meteorological station of the INTA Manfredi (Instituto Nacional de Tecnología Agropecuaria Manfredi, Córdoba, Argentina) for 31°49′ South latitude; 63°46′ West Length, a 292 masl), which is located 60 km SE from Córdoba city (https://inta.gob.ar/documentos/informacion-meteorologica-mensual-de-la-eea-manfredi).

### Data analysis to verify periodicity in biological and meteorological parameters

Two different mathematical approaches (autocorrelation and wavelet analysis) were applied) to detect periodicity and precise evolution time in sperm physiology variations.

#### Autocorrelation

This math analysis generates correlation coefficients for the time series between the biological data and itself as it is sequentially ‘lagged’ out of phase, one time unit at a time. An autocorrelation function C(s) is estimated between two points in time with a determined lag (s), meaning that data from the same variable at a given time are correlated with data from the same variable taken at a later time. A periodic behavior is distinguished from a random one according to the correlation coefficients robustness and the shape of the curve^50,51^. Thus, recurring peaks in the correlation curve (meaning positive values followed by negative values and again by a positive one) indicate that the signal is periodic. Conversely, a random behavior (linear stochastic processes) is also detected by the autocorrelation function, which is represented by an exponential decay exhibiting correlation coefficients approximating to zero. Data analysis was performed using MATLAB R2017a.

#### Wavelet analysis

This analytical approach consists in a mathematical transformation of a time series of biological data according to a complex mathematical function, which provides information simultaneously on the presence of periodic behavior and its time localization^52–55^. Wavelet complex analysis expresses a signal (in this case a time series) as a sum of its component waveforms (real and imaginary parts) (Supplementary Fig. S6). Here, the continuous wavelet transform function coefficient (*cwt)* of the time series is represented by their real part (dotted line in Fig. 6 and Supplementary Fig. 6). In the process of analysis, this wavelet is rescaled for each time scale (period) evaluated (“Y” axis in Supplementary Fig. S5). Here we used two complex wavelet functions, the Morlet and the Gaussian. Data analysis in this article was performed using the wavelet toolbox of MATLAB R2017a, in particular the continuous wavelet transform function. The complex Morlet wavelet, cmor1–1.5, was used with scales that ranged from 0.3 to 30, corresponding to periods of 0.2 to 20 months^55,56^. The complex Gaussian wavelet function (cgau1), was used with scales that ranged from 0.03 to 6, corresponding to periods of 0.02 to 20 months. For convenience, scales were transformed into frequencies using the scales2freq function of MATLAB. The developed script is publicly available^57^. For visualization of synchronicity, we followed Pering *et al*.^54^ using the real part of the cwt (Re(cwt)), in which the Spearman correlation between Re(cwt) of the different signals is evaluated at a given time scale (for review see Kurz *et al*., 2017)^58^.

### Statistical analysis

Data were expressed as the mean ± SEM of three to ten independent experiments. Differences between treatments were determined by means of one-way ANOVA, and a posteriori Tukey test performed with the Graph Pad Prism 6.01 (La Jolla, CA, USA) unless otherwise indicated, considering statistically significant differences at a level of confidence of 0.05. Cytometry data were analyzed automatically using FACS Diva and FlowJo software and the statistical analysis was performed using the RStudio software, v1.0143^59^. All data were verified to satisfy the parametric assumptions of homogeneity of variances and normality. Statistical approach for time series analysis is described in the previous section.

## Supplementary information

Supplementary Information
