# Supplementary material for: Sperm physiology varies according to ultradian and infradian rhythms
Source: Sci Rep. 2019 Apr 12;9:5988. doi: 10.1038/s41598-019-42430-4 (PMC6461627; doi:10.1038/s41598-019-42430-4)
Supplement: Supplementary file 1 — Supplementary Information [file 41598_2019_42430_MOESM1_ESM.pdf]

Sperm physiology varies according to ultradian and infradian rhythms

Ayelén Moreno-Irusta [1,2], Jackelyn M. Kembro [2], Esteban M. Domínguez [1,2], Arturo Matamoros-Volante [3], Maria N. Gallea [1,2], Rosa Molina [4], Hector A. Guidobaldi [1,2], Claudia L. Treviño [3], Maria J. Figueras [1,2], Ana Babini [5], Nelso A. Paina [6], Carlos A.N. Mercado [6], Laura C. Giojalas [1,2*].

[1]Universidad Nacional de Córdoba (UNC), Facultad de Ciencias Exactas, Físicas y Naturales, Centro de Biología Celular y Molecular, Córdoba, Argentina. [2]Instituto de Investigaciones Biológicas y Tecnológicas, UNC, CONICET, FCEFyN, Córdoba, Argentina.[3]Departamento de Genética del Desarrollo y Fisiología Molecular, Instituto de Biotecnología, Universidad Nacional Autónoma de México, México, [4]Laboratorio de Andrología y Reproducción (LAR), Córdoba, Argentina. [5]Universidad Nacional de Córdoba, Facultad de Ciencias Médicas, Instituto Universitario de Medicina Reproductiva (IUMER), Córdoba, Argentina; [6] Universidad Nacional de Córdoba, Hospital Universitario de Maternidad Nacional, Córdoba, Argentina.

*Corresponding author; E-mail: lgiojalas@unc.edu.ar

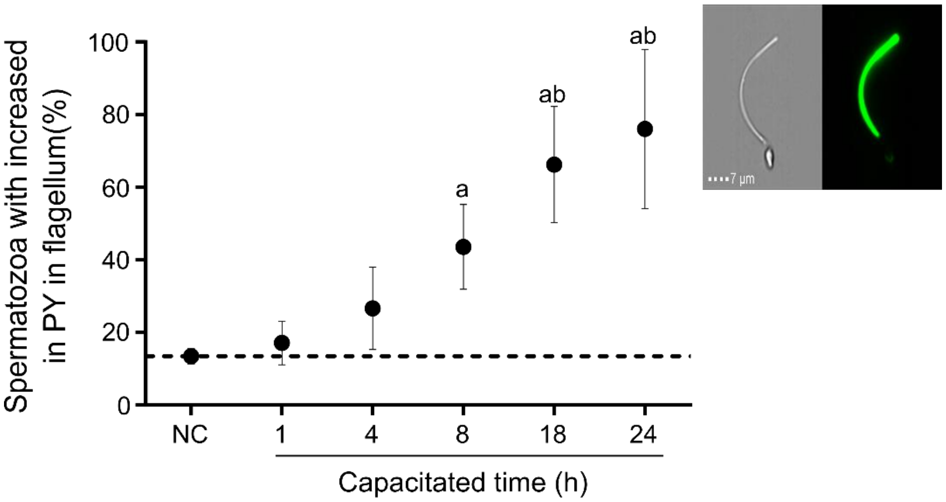

Supplementary Figure S1: Capacitated spermatozoa determined as the percentage of spermatozoa with PY labeling in the flagellum. Sperm cells were incubated under non-capacitating conditions (NC) or capacitated for 1, 4, 8, 18 or 24 hours. Each dot represents the median value in each condition (n= ~2000 cells per donor). For the analysis, the percentage of spermatozoa exceeding the third quartile of fluorescence intensity was taken; the dotted line shows the percentage of spermatozoa that exceeds the third quartile of fluorescence intensity in the non-capacitated samples (NC). The inset shows a representative phase contrast and fluorescence images of a representative spermatozoon, capacitated for 18 hours. Data are ae mean±SE from 7 independent experiments performed with ejaculates from different donors. [a] Statistically significant differences vs NC treatment. [b] statistically significant differences vs 8 h.

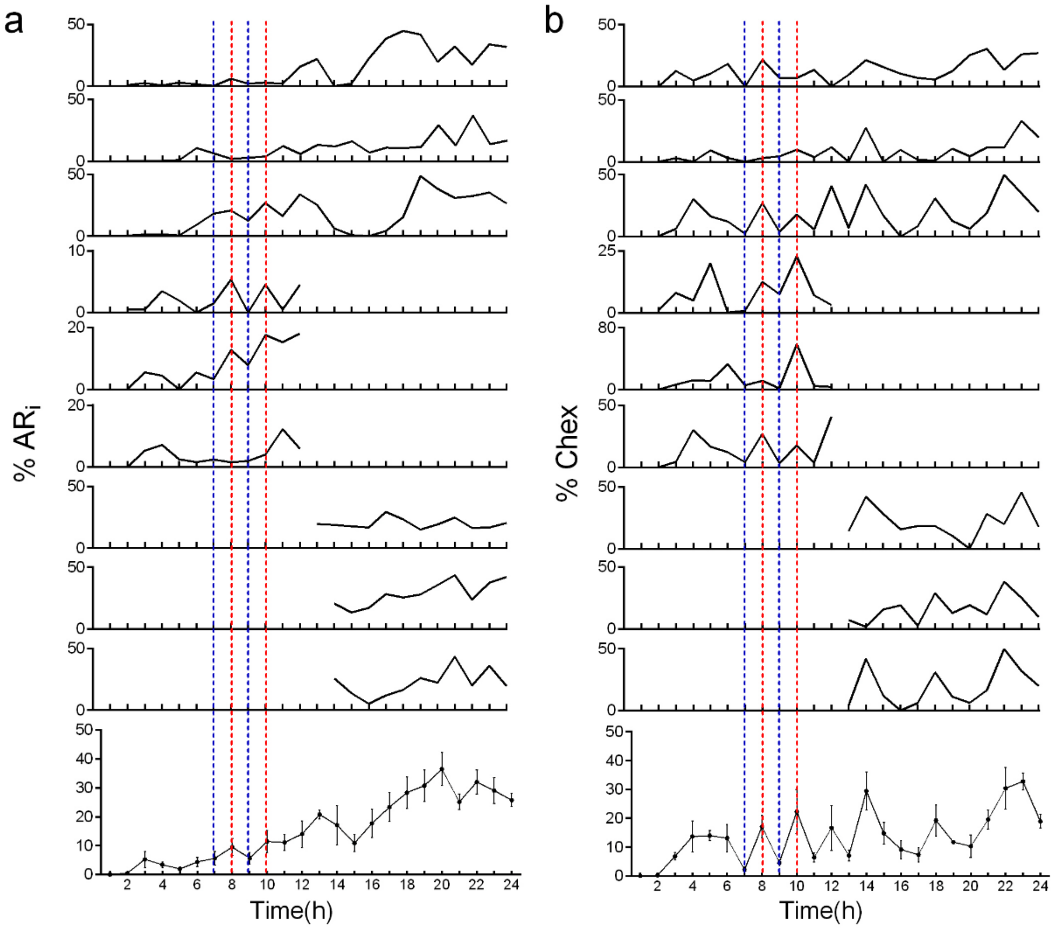

Supplementary Figure S2: Individual variation of human semen samples along incubation time. The percentage of AR_i **(A)** and % Chex spermatozoa **(B)** are shown. The bottom panel shows the average for each hour shown in Figure 1. Vertical blue and red lines show valleys and peaks, respectively. ARi, induced acrosome reaction; Chex, capacitated spermatozoa recruited by chemotaxis.

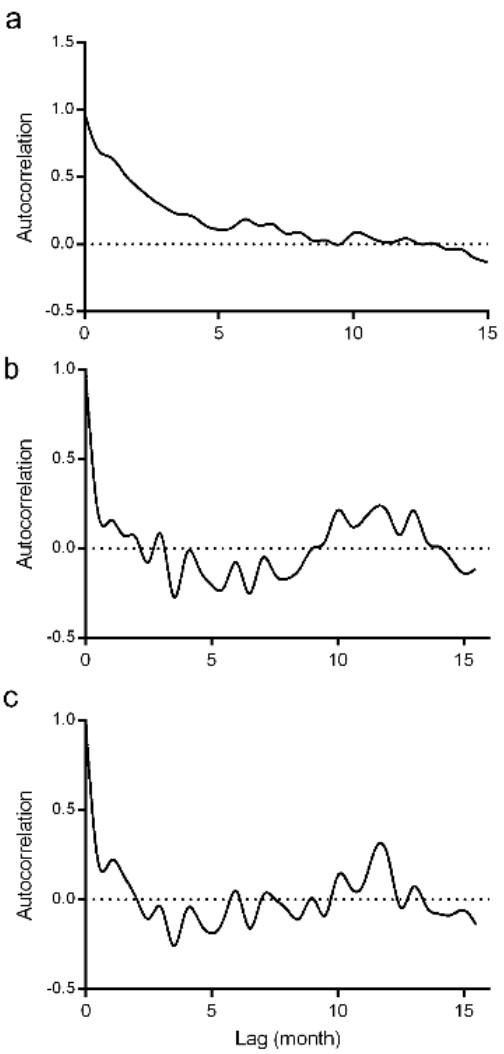

Supplementary Figure S3: Autocorrelation analysis for conventional sperm parameters over a 3-year period. Time series from data shown in Figure 5. Sperm concentration **(A)**, motility **(B)** and morphology**(C)**.

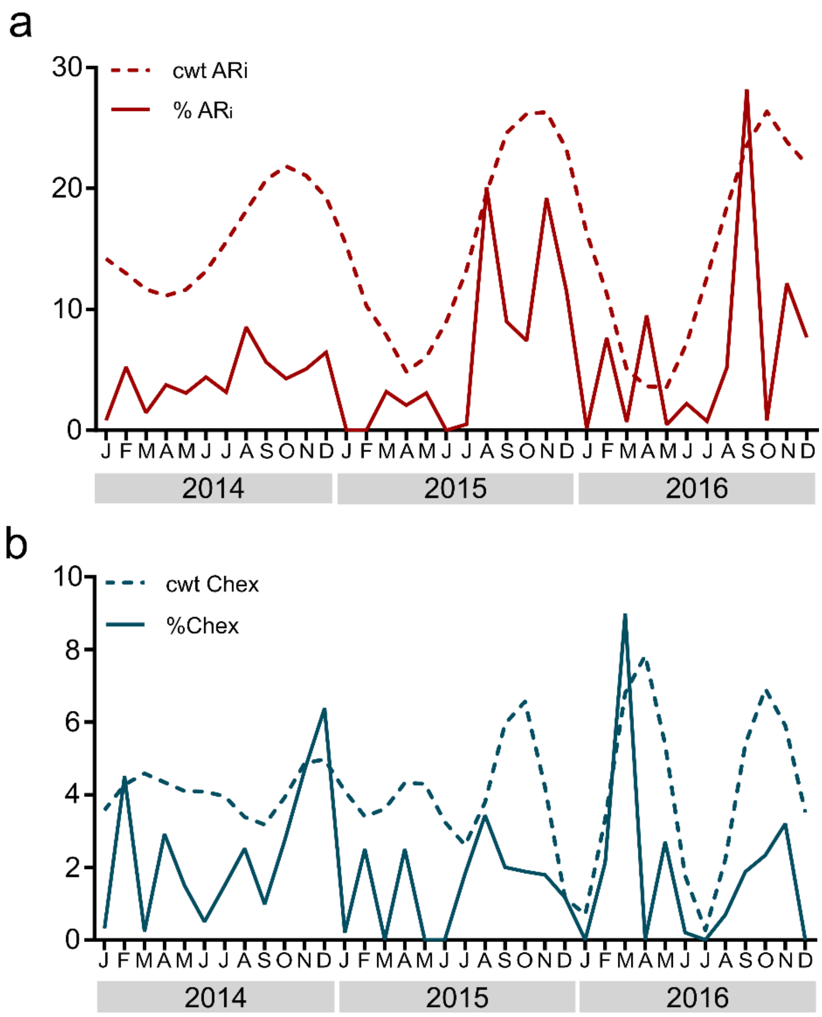

Supplementary Figure S4. Infradian rhythms observed in sperm physiology after 4 h incubation. The time series of the % ARi sperm and the % Chex sperm are shown in panel A and B, respectively. The corresponding Morlet Wavelet coefficients (cwt) at different monthly scale (dotted lines) are shown in each panel. Data are expressed as the mean of 3 - 4 independent experiments, for each month, performed with ejaculates from different donors. ARi, induced acrosome reaction; Chex, capacitated spermatozoa recruited by chemotaxis; cwt, wavelet coefficients.

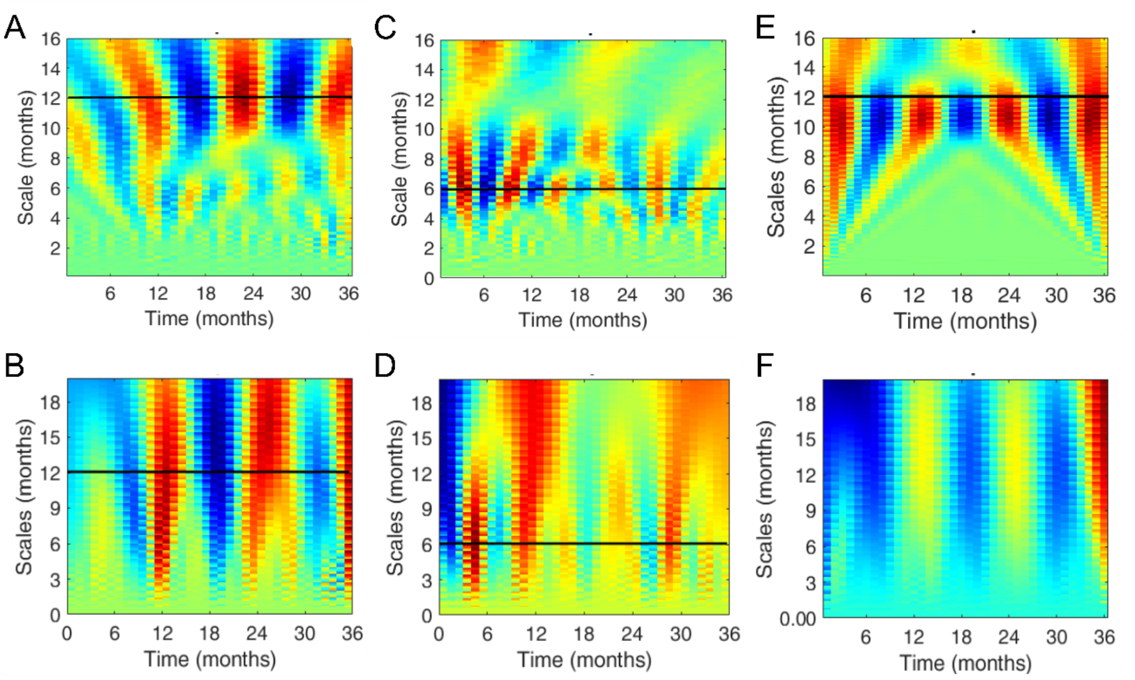

Supplementary Figure S5: Wavelet analysis of time-series for sperm capacitation and meteorological parameters. Amplitude plots of complex Morlet coefficients (Real part) of %ARi (A). The x-axis represents time (3 years) and the y-axis indicates the scale of the wavelet used (from 0.3 to 16 months). The wavelet decomposition shows a clear 12-month cycle, as marked by the black line, which coincides with Autocorrelation Analysis shown in Fig. 6A. Similar results were observed using a Gaussian wavelet (B). Amplitude plots of complex Morlet coefficients (Real part) of % Chex (C). The wavelet decomposition shows a clear 6-month cycle, as marked by the black line, which coincides with the autocorrelation analysis shown in Fig. 6B. Similar results were observed using a Gaussian wavelet (D). Amplitude plots of complex Morlet coefficients (Real part) of Photoperiod (E) and Temperature (F). The wavelet decomposition shows a clear 12-month cycle, as marked by the black line, which coincides with Autocorrelation Analysis shown in Fig. 6C.

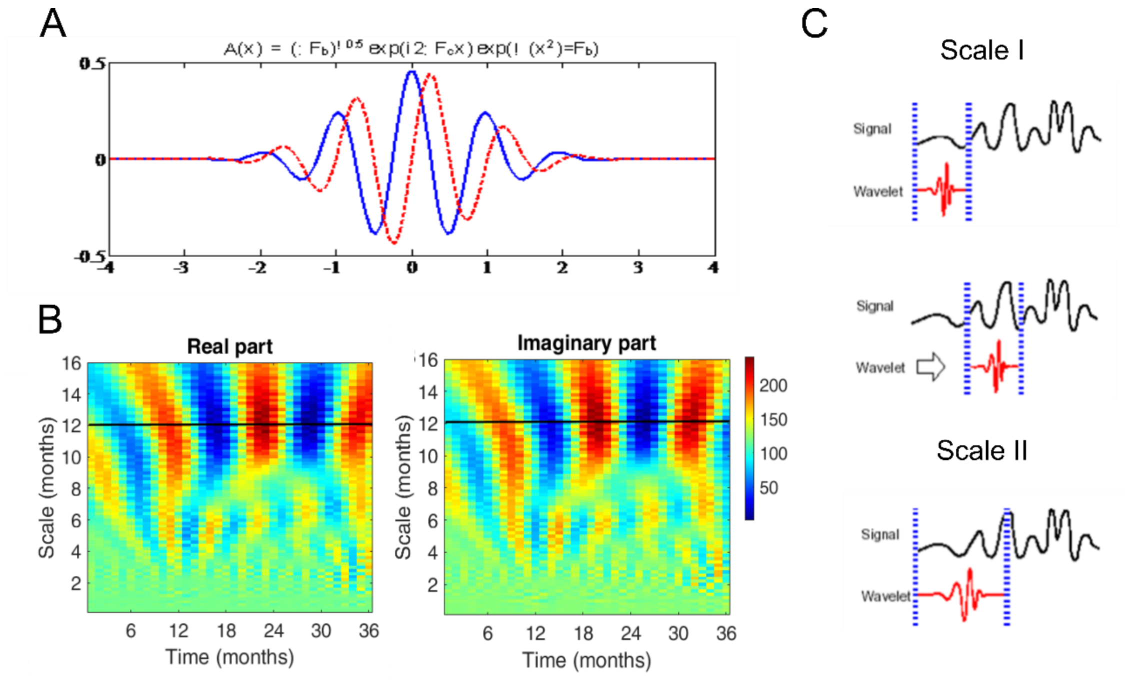

Supplementary Figure S6: Features of the wavelet analysis. Example of the shape of the Morlet wavelet.Real (solid blue line) and imaginary (dashed red line) part of the Morlet wavelet with parameters bandwidth (A). Example of amplitude plots of complex Morlet coefficient: Real (left) andImaginary part (right). The x-axis represents time (3 years) and the y-axis indicates the scale of the wavelet used (from 0.3 to 16 months) (B). Schematic diagram of how wavelet analysis works (C).
